# Mastocytosis: Fertility and Pregnancy Management in a Rare Disease

**DOI:** 10.3389/fonc.2022.874178

**Published:** 2022-04-27

**Authors:** Jacqueline Ferrari, Pietro Benvenuti, Elisa Bono, Nicolas Fiorelli, Chiara Elena

**Affiliations:** ^1^ Department of Molecular Medicine, University of Pavia, Pavia, Italy; ^2^ Division of Hematology, Istituto di Ricovero e Cura a Carattere Scientifico (IRCCS) S. Matteo Hospital Foundation, Pavia, Italy

**Keywords:** cutaneous mastocytosis, systemic mastocytosis, myeloid neoplasms, pregnancy, multidisciplinar management

## Abstract

Mastocytosis encompasses a subset of rare diseases, characterized by the presence and accumulation of abnormal neoplastic MC in various organ systems, including skin, bone marrow, spleen and gastrointestinal tract. Clinical manifestations are highly heterogeneous, as they result from both MC mediator release and MC organ infiltration. Both pregnancy, a lifetime dominated by huge physiological changes, and labor can provide triggers that could induce worsening of mastocytosis symptoms. On the other hand, mastocytosis has relevant implications in obstetric management and prenatal care during all the pregnancy. In this review article, current knowledge about the impact of mastocytosis on fertility and pregnancy outcome will be reviewed and discussed, with the aim to provide clinical practice guidance for the evaluation and management of pregnancy and delivery in patients with cutaneous and systemic mastocytosis.

## Introduction

Mastocytosis encompasses a subset of rare diseases, characterized by the presence and accumulation of abnormal neoplastic mast cells (MC) in various organ systems, including skin, bone marrow, spleen and gastrointestinal tract. The recently updated WHO classification (1) identifies three different entities: Cutaneous Mastocytosis (CM), Systemic Mastocytosis (SM) and Mast Cell Sarcoma (MCS). CM is more frequent in infancy and childhood, and is characterized by dermal infiltration of MC, without involvement of any other organ or system. It presents with peculiar skin lesions, and could be associated with urticaria, pruritus, flushing and dermographism. The diagnosis of SM relies on the histological confirmation of extra-cutaneous involvement from neoplastic MC, and 30% of patients do not present skin manifestations. The disease burden and the presence of organ damage, whose entity is defined according to B and C findings (1), together with the identification of an associated hematologic malignancy different from SM, lead to classify SM in Indolent SM (ISM), Smoldering SM (SSM), Aggressive SM (ASM), SM with an associated hematologic neoplasm (SM-AHN) and mast cell leukemia (MCL) (1). The clinical manifestations are highly heterogeneous, resulting both from MC mediator release and MC organ infiltration.

Mastocytosis is associated in most cases with somatic gain-of-function point mutations of the c-*KIT* gene. KIT (CD117) is a tyrosine kinase receptor expressed by MC, and it plays a relevant role in regulating normal MC proliferation, maturation, adhesion, chemotaxis, and survival (2, 3). Gain-of-function somatic mutations in *KIT* gene, in particular the D816V mutation, are identified in the majority of cases of adult SM, irrespective of SM subtype (4). More recently, biological studies have identified germline or acquired activating KIT mutations also in childhood-onset mastocytosis, confirming its clonal nature (5).

Patients with CM and ISM are mainly treated with anti-mediator drugs, including histamine H1 and H2 antagonists, antileukotriene agents, and sodium cromolyn. Advanced forms of SM can require cytoreductive treatment: current treatment options could include alfa-interferon, cladribine and tyrosine kinase inhibitors (mostly Midostaurin, Imatinib and Avapritinib), and, in selected cases, stem cell transplantation (4). After the increased availability of TKIs, alfa interferon is less frequently used, due to its slow onset of efficacy and poor tolerability; however it may still be the safest option in pregnant patients that require cytoreductive treatment.

The prevalence of the disease in the general population, due to its rarity, is not completely established; in general, median age of onset is considered to be lower than that of other hematologic malignancies, making conception and pregnancy a potential occurrence during the patient’s life.

Both pregnancy, a lifetime dominated by great physiological changes, and labor can provide many triggers that could induce worsening of mastocytosis symptoms. On the other hand, mastocytosis has relevant implications in obstetric management and prenatal care during all the pregnancy. There is limited information about the impact of mastocytosis on pregnancy: the available knowledge is mainly derived from single case reports/retrospective case series with small number of patients with ISM/CM, and prospective data are lacking.

In this review article, the impact of mastocytosis on fertility and pregnancy outcome and the management of mastocytosis during pregnancy and delivery will be reviewed and discussed.

## The Physiological Role of Mast Cells in Reproductive Function and Pregnancy

MC play a key role in inflammatory reactions in allergic and non-allergic processes; although the most known trigger is the cross-linking of Fc IgE receptors, responsible of MC activation during immediate hypersensitivity reactions, also other stimuli, including aggregated IgG (6), complement proteins (7), peptides (8), and sexual hormones (9) are involved in MC activation and regulation.

MC are present in the endometrium in all stages of the menstrual cycle, with similar MC numbers in the different layers (functionalis, basalis, and muscularis). A study evaluating endometrial biopsies at different time points during the menstrual cycle documented changes in MC morphology, granule content and an extensive MC activation/degranulation in the functional layer, as derived by extracellular tryptase, just prior to and during menstruation; extracellular MC tryptase and chymase are able to activate the matrix metalloproteinases, enzymes involved in stromal degradation. This observation could explain the premenstrual exacerbation of allergic symptoms, asthma and atopic dermatitis in affected women (10).

In the same way, the migration of MC to the uterus in the first stages of pregnancy is regulated by estradiol and progesterone changes. These cells, with the degranulation of histamine, metalloproteinases, tryptase and vascular endothelial growth factor (VEGF), are known to be involved in the embryo’s attachment and implantation and the subsequent placentation into the endometrium. Additionally, MC degranulation correlates with angiogenesis, remodelling and spiral artery modifications throughout pregnancy (11).

MC density has been documented to be significantly higher in myometrium in pregnant women than in non-pregnant women, and systemic MC activation due to allergen exposure in sensitized patients was associated with an immediate and substantial increase in myometrial contraction (12). Moreover, a significant role of serum histamine and other MC mediators in increasing myometrium contractility *in vitro* in mice, guinea pigs and humans during pregnancy has been reported, and inhibition of this effect by MC stabilizers and antihistamines has been documented (13–17). Taken together, these observations indicate a possible role for MC in mediating uterine contractility in pregnancy and risk of preterm labor.

Some cases of recurrent breastfeeding anaphylaxis in healthy women have been reported, suggesting that, also after delivery, hormonal changes could induce MC activation and degranulation (18, 19).

Collectively, all these data support the hypothesis that MC are strongly involved in the human reproductive function, and that the physiological changes that occur during pregnancy could significantly affect MC activity. Conversely, there are few data about the behaviour and the functional modifications of neoplastic MC during pregnancy in women with SM, and prospective studies are warranted.

## MC-Mediator Release Related Symptoms During Pregnancy in Mastocytosis

Mastocytosis-related symptoms reported during pregnancy include skin manifestations, such as flushing, urticaria and pruritus, gastrointestinal symptoms, and anaphylaxis (20–22). The evolution of MC-mediator release-associated symptoms during pregnancy can be highly variable, as they have been reported to improving, to remaining stable, or significantly worsening. In a small series of patients reported by Bruns et al, 4 out of 12 women during pregnancy experienced a deterioration of mastocytosis symptoms, 7 patients reported no change, and one patient observed an improvement (23). In a Spanish cohort reported by Matito and colleagues, worsening of symptoms was observed in only 22% (10 of 45) of cases, whereas they were stable in 45% and improved in 33% of pregnancies. Interestingly, the improvement and resolution of symptoms were observed during the first trimester and lasted throughout the whole pregnancy (20).

A worsening of MC mediator related symptoms during pregnancy was observed in a similar percentage of patients (4 of 17, 24%) by Polish investigators (21), whereas Worobec et al. observed an exacerbation of symptoms in 63% of cases (22). Moreover, in this report the authors described a significant increase in the skin lesions in almost all the patients (3/8 during pregnancy, and 4/8 early after delivery) (22), whereas in the above mentioned Spanish series skin changes were observed in only one patient that reported worsening of skin infiltration (20).

Overall, these clinical observations suggest that hormonal and immunological modifications occurring during pregnancy may have a potential effect on MC activation *in vivo*. In addition, an increase of circulating interferons secreted by the placenta during gestation may also affect MC activity. However, clinical and biological data prospectively collected are required to substantiate this hypothesis.

The significant discrepancy in the trend/evolution of symptoms among different reports could be related to the relatively limited number of cases, the heterogeneity of SM subtypes (ISM vs CM), and the different methods used to retrospectively collect clinical information during pregnancy. Moreover, it is not infrequent that antimediator treatments are irregularly taken or reduced during pregnancy, due to safety concerns. The reduction in medication and a discontinuous drug intake could be associated with the variability of mastocytosis symptoms.

Nevertheless, it is important to underline that in about half of the cases the clinical changes occurred during pregnancy lasted also after delivery, therefore a certain causative role of the gestational status cannot be definitively established.

## Occurrence of Anaphylaxis During Pregnancy in Mastocytosis

Anaphylaxis is a potentially life-threatening event for both the mother and the foetus. The risk of anaphylaxis during delivery in the general population is approximately 2.7 per 100,000 deliveries (24, 25). Signs and symptoms of anaphylaxis in pregnancy may include diffuse pruritus, gastrointestinal symptoms, lower back pain, myometrial contractions, preterm labor, and vulvar or vaginal itching (24, 26). Anaphylaxis may result in maternal hypotension, which can lead to a decreased uterine blood flow and foetal hypoxiemia, and in turn to fetal injury, including severe central nervous system damage, hypoxic-ischemic encephalopathy, or death. The risk of cesarean delivery in anaphylaxis is significantly elevated, as high as 74% (24–26).

The occurrence of anaphylaxis during pregnancy in patients with mastocytosis has been reported only in the Spanish series (4 of 45 patients, 8%): in one case it was triggered by Hymenoptera sting, whereas it was of idiopathic origin in other three cases. In none of these cases uterine contractions or signs of preterm labor were reported during anaphylaxis, and symptoms resolved with H1 antihistamines and/or corticosteroids, without epinephrine administration. Interestingly, in 2 of 4 patients with recurrent idiopathic anaphylaxis in the pregestational period, no anaphilactoid episodes were registered during pregnancy (20).

Watson et al. described the case of a woman with a drug-associated (terbutaline) severe hypotension occurred at 31 weeks of gestation, that resulted in fetal death. In this woman, SM diagnosis was subsequently made due to the persistency of elevated levels of serum tryptase. Her following pregnancy was effectively managed by a multidisciplinary team, and the patient was treated with antihistamine prophylaxis for the whole gestation. Despite the sudden occurrence of severe hypotension during delivery, that requested steroids and epinephrine therapy, a healthy baby was delivered without other maternal complications (27).

Some cases of recurrent breastfeeding anaphylaxis in healthy women have been previously reported (18, 19), but to date there are no data about safety of breastfeeding in mastocytosis patients.

## Pregnancy Outcomes

### Fertility Rate and Spontaneous Miscarriage

The diagnosis of SM does not globally appear to have any effect on fertility, although there are very few data and prospective evaluation in terms of ovarian function and fertility potential of young women affected by SM are lacking. All conceptions reported in the literature have occurred spontaneously without procedures of medically assisted procreation apart from one, achieved by *in vitro* fertilization due to a history of infertility (20). Worobec et al. described a pre-existing gynecologic disease potentially associated with reduction of fertility (primary ovarian failure and endometriosis) in 3 out of 9 (33%) women with ISM, a rate similar to that of the general population. Only one of these three women was treated with clomiphene to help pregnancy achievement, the other two women being able to conceive spontaneously (22). Similarly, in the above mentioned Polish cohort all the conception occurred without any medical intervention (21).

Whether mastocytosis may result in significantly increased rates of spontaneous miscarriage is unclear. The Polish study found that the rate of spontaneous abortion was slightly higher in a cohort of mastocytosis patients than reported in the general population (25-30% vs 8-20%) (21, 28, 29). Similarly, Worobec et al. reported miscarriages in the first trimester in 2 out of 8 (25%) women (22), whereas only 6/51 (11%) pregnancy losses were reported in the cohort described by Matito et al. with a frequency not significantly different from the general Spanish population (20, 28).

### Preterm Labor and Birth

It has been described that elevated histamine levels, with consequent increased formation of gap junctions in myometrium, may results in uterine myometrial contractions *in vitro*, but whether this could be associated with an increased risk of preterm labor in mastocytosis patients is still unknown.

Three clinical cases (30–32) reported onset of preterm labor at 24-27 weeks, associated with high level of serum and urinary histamine: delivery was successfully reached at 39, 36 and 40 weeks, respectively, and in two of these cases tocolytics and antihistamines were requested to control premature uterine contractions. In the Polish study 4/23 (17%) pregnancies resulted in preterm birth, including one delivery at 26^th^ week of gestation due to onset of preeclampsia, and three deliveries between 36 and 37 week of gestation, without other pregnancy complications. In two cases preterm labor resulted in a fullterm health baby without need of specific therapy (21). Conversely, the studies by Matito and Worobec did not report significant detrimental effects of mastocytosis on pregnancy outcome (20, 22). In the Spanish cohort only 3 out of 45 births (6.6%) were preterm, and this percentage is similar to the European rate (5%) and high-income country rate (8%) of preterm birth (20, 33) Interestingly, the four pregnant women that experienced anaphylaxis in this cohort did not develop preterm labor.

### Other Pregnancy Complications

No other significant recurrent pregnancy complications have been so far reported. Published data include: a) one case of preeclampsia not complicated by preterm birth (22), b) one case of severe anemia due to excessive bleeding after cesarean delivery (22), c) one case of pregnancy-associated hypertension (21), d) one case of deep venous thrombosis (21), e) one toxoplasmosis infection during pregnancy (21). The sporadic occurrence of all these events suggest that they are likely independent from the underlying mastocytosis, and therefore should be managed according to obstetric guidelines. No stillbirths have been registered so far.

## Labor Management and Outcome

The time of labor and delivery represents a crucial event, potentially associated with severe complications. In order to reduce the potential risk of any complication, the labor and delivery period should be managed by a multidisciplinar team of experts in the field of mastocytosis.

Information on management of labor and delivery is limited and is derived from literature on peri−/intra-operative management in mastocytosis. The type of delivery (vaginal or cesarean) do not seem to affect the outcome, and the choice of the procedure can rely on obstetrical indications and patient’s preference. In the series reported by Matito and colleagues, 78% (35/45) of cases ended with a vaginal delivery, based on obstetric indications, whereas only in 22% (10/45) of cases a cesarean birth was performed. No relationship between the onset of complications and the type of labor was reported (20). Similarly, in the Polish cohort there was a prevalence of vaginal delivery (12/17 women, 70%), while a cesarean section was performed in 5 cases, due to both obstetric and hematologic reasons, with epidural analgesia and without any complication of labor and delivery (21).

Labor induction using both oxytocin and dinoprostone was carried out in a minority of patients and was reported to be safe and effective in all the cases (20, 22, 32).

The risk of a severe degranulation reaction is significantly increased during labor and delivery in comparison with the whole pregnancy, due to anesthetic procedures, other drugs eventually used, and the labor stress itself. Peripartum prophylactic antimediator treatment, including different combinations of oral and intravenous H1 and H2 antihistamines and steroids, according to each anesthesiologist’s criteria, has been demonstrated to be effective and free from any side effects. MC degranulation symptoms (typically skin symptoms, including pruritus, generalized erythema, and flushing) were observed in a minority of cases (5/45 pregnancies in the Spanish cohort) (20), during or immediately after delivery, in particular in patients who did not undergone any antimediator prophylaxis. No life-threating events were observed in all the pregnancies reported in literature; in one case a patient experienced an excessive bleeding immediately after cesarean delivery, that required a blood transfusion (22). The few others case series suggest similar findings, with no increased risk of complications of labor and delivery.

Resuscitation equipment (anti H1 e H2, steroids, epinephrin) should be available during labor, delivery and in the early post-partum period. In addition, the active medical observation after delivery should be longer than in healthy women: SM patients should not be left alone immediately after partum.

## Management of Drugs During Pregnancy and Labor

During pregnancy a significant proportion of patients needs to continue medications in order to control MC mediator related symptoms. Worobec et al. reported that approximately half of the women were treated with antimediators, including H1 antihistamines, and less commonly, oral prednisone, in order to control mastocytosis symptoms (22). On the other hand, in the series reported by Ciach et al. only 5/17 women needed H1 and H2 antihistamines during pregnancy (21).

There are safety concerns regarding theratogenic potential of drugs used during pregnancy. Actual guidelines recommend using drugs with a known safety profile, according to FDA risk category. The available knowledge about safety in pregnancy and lactation of most of the drugs frequently used in mastocytosis is summarized in **Table 1** as reported by NCCN guidelines (34, 35): clorpheniramine, cetirizine, loratadine, ranitidine and famotidine can be used, whereas oral disodium cromolyn, desloratadine and ketotifene should be discontinued (34).

**Table 1:** Mastocytosis treatments and pregnancy/lactation risk.

Group	Medication	FDA Risk Category	Crosses placenta	Pregnancy implications	Excreted in breast milk	Lactation
First-generation H1 antihistamines	Chlorpheniramine	B		No increased risk of birth defects	Yes	Use with caution
	Dimenhydrinate	B	Yes	No increased risk of fetal abnormalities	Yes	Use with caution
	Diphenhydramine	B	Yes	Unclear, historical association with cleft palate	Yes	Breast-feeding contraindicated
	Doxylamine	A		Historical association with neural tube defects, oral clefts, hypoplastic left heart	Yes	Breast-feeding not recommended
	Hydroxyzine	C	Yes	No increased risk of birth defects but not recommended in early pregnancy	Unknown	Breast-feeding not recommended
	Meclizine	B		No increased risk of birth defects	Unknown	Use with caution
Second-generation H1 antihistamines	Cetirizine	B		No increased risk of birth defects	Yes	Monitor infants for drowsiness, irritability
	Levocetirizine	B		No increased risk of birth defects	Unknown	Breast-feeding not recommended
	Loratadine	B		No increased risk of birth defects; prior historical association with hypospadias	Yes	Metabolites present in breast milk
	Fexofenadine	C		Limited information available	Yes	Limited information available
	Desloratadine	C		Adverse events in animal studies	Yes	Limited information available
H2 antihistamines	Cimetidine	B	Yes	No increased risk of birth defects	Yes	Breast-feeding not recommended
	Famotidine	B	Yes	No increased risk of birth defects	Yes	Use with caution
	Ranitidine	B	Yes	No increased risk of birth defects	Yes	Use with caution
Mast Cell stabilizer	Cromolyn	B		Safe in pregnancy	Unknown	Use with caution
	Ketotifen	C		Adverse events in animal studies	Unknown	Breast-feeding not recommended
Anti-IgE antibody	Omalizumab	B		No increased risk of birth defects	Unknown	IgG excreted into breast milk; not recommended
Glucocorticoids	Hydrocortisone	C		Increased risk of oral clefts with use in first trimester	Yes	Wait 4 hours after dose
	Prednisone	C	Yes	Increased risk of oral clefts with use in first trimester	Yes	
	Betamethasone	C	Yes	Increased risk of oral clefts with use in first trimester; non-fluorinated corticosteroid preferred	Yes	Wait 4 hours after dose
	Dexamethasone	C	Yes	Increased risk of oral clefts with use in first trimester; non-fluorinated corticosteroid preferred	Yes	Wait 4 hours after dose
Leukotriene receptor antagonist	Montelukast	B		No increased risk of birth defects	Yes	Use with caution
Cytoreductive therapies	Cladribine	D		Teratogenic effects and fetal mortality observed		Not recommended
	Imatinib	D	Yes	Pregnancy not recommended (in mother or father) within 2 weeks of last dose	Yes	Not recommended
	Midostaurin	Not assigned		Pregnancy not recommended (in mother or father) during or within 4 months of last dose	Unknwon	Not recommended
	Interferon alpha 2b	C	No	No clear association	Yes	

Modified from Lei et al. NCCN Version 2.2019.

FDA Risk categories: A = adequate and well-controlled studies have failed to demonstrate a risk to the fetus in the first trimester of pregnancy (and there is no evidence of risk in later trimesters). B = animal reproduction studies have failed to demonstrate a risk to the fetus and there are no adequate and well-controlled studies in pregnant women. C = animal reproduction studies have shown an adverse effect on the fetus and there are no adequate and well-controlled studies in humans, but potential benefits may warrant use of the drug in pregnant women despite potential risks. D = there is positive evidence of human fetal risk based on adverse reaction data from investigational or marketing experience or studies in humans, but potential benefits may warrant use of the drug in pregnant women despite potential risks.

Dosage of antimediator treatment should be titrated in order to identify the smallest effective dose to minimize potential harm to the foetus; in the cases reported in literature dosages were often decreased due to fetal health concerns, and this point should be taken into consideration when analyzing the evolution of symptoms throughout the pregnancy. Interestingly, Lei et al. reported the case of a 35-year-old woman with highly symptomatic ISM, with frequent and severe episodes of idiopathic anaphylaxis, who was finally treated with interferon (IFN) alfa-2b, after previously failing multiple lines of treatment, including antihistamines, cromolyn, ketotifen and hydroxyrurea. After multidisciplinar counseling with the maternal-fetal medicine team, she was allowed to realize her desire of conception and carry out her pregnancy continuing IFN, together with antihistamines and leukotriene antagonists. Although she had fluctuating symptoms during the course of the pregnancy, that needed a stable increase in the IFN dose and occasional boosters of steroids, she finally gave birth to a healthy child (35).

No limitations about local, epidural and general anesthesia emerged from the literature, if carried out according to consensus guidelines about management of anesthetics in SM (36, 37).

## Infant’s Health

Detailed information about newborns delivered by women affected by SM is very scarce, but apparently there are not significant concerns about infant’s health.

One study reported a significant proportion of low birth weight infants (weight in the 10th percentile), (3/8 infants, 38%), not associated with any evident complication occurred during the pregnancy (22). In one case, a baby was born with hydrocephalus and later developed a developmental delay. In this study no subjects with urticaria pigmentosa or systemic mastocytosis were identified, neither at birth, nor during follow-up (some cases followed up to 50s years old).

In the Polish cohort, three out of 17 (17%) newborns had low birth weight infants: the one born at 26 weeks of gestation due to preeclampsia subsequently died because of prematurity complications; in one case Patau’s syndrome was diagnosed. One baby presented cutaneous mastocytosis at birth, but information about persistence or resolution of mastocytosis with child’s growth is lacking (21).

In the series reported by Matito et al., 4/45 were LBW (8%), three of whom associated with full term pregnancies. One newborn was diagnosed with Down’s syndrome. One child developed CM several years after birth; this child is a 12-year-old female who refused a BM study (20). Familial mastocytosis is a rare, but well known entity, especially in well-differentiated systemic mastocytosis (WDSM), and this case underlines the importance to extend the follow-up of children of women with SM as long as possible, to collect more information and improve diagnosis and management.

## Critical Points in the Clinical Management of Pregnancy and Delivery in Mastocytosis

The overall frequencies of spontaneous pregnancy loss, preterm birth, low birth weight and other obstetric complications seem not to significantly differ from the general population. Therefore, despite the limited available information, it may be concluded that non-aggressive categories of mastocytosis do not seem to have a relevant impact on the outcome of pregnancy and that women with mastocytosis should not be advised against pregnancy. The key points of the current knowledge about pregnancy and mastocytosis are summarized in **Table 2**.

**Table 2:** Managing mastocytosis and pregnancy: key points.

Key points
A diagnosis of SM mastocytosis does not appear to negatively affect fertility There are no sufficient data to determine whether a diagnosis of mastocytosis could result in an increased rate of adverse maternal or fetal outcome compared to the general population. No severe or life-threating complication have been observed up to now, suggesting that patients with nonaggressive categories of mastocytosis should not be advised against pregnancy. A multidisciplinary team, including high-risk obstetricians, haematologists, allergists and anesthesiologists, should be involved in the clinical management throughout the whole pregnancy, from the pre-conception period to the postpartum Mastocytosis shows a heterogeneous clinical behavior during pregnancy, as clinical manifestations and symptoms could worsen, remain stable, or improve. Allowed antimediator drugs should be titrated to control MC mediator release symptoms and minimize potential harm to the foetus Anesthetic drugs can be safely given to pregnant mastocytosis patients

The patient should be referred to a reference center, and managed by a mastocytosis-dedicated multidisciplinary team, including a high-risk obstetrician, an hematologist, an allergist and an anesthesiologist.

It is of outmost importance to collect a detailed survey of the patient’s prior hematologic and obstetric history, including information about previous pregnancies or history of infertility, mastocytosis subtype and activity, previous anaphylaxis, and known drug allergies.

During pregnancy, regular visits should be planned, to accurately evaluate symptoms’ evolution and to optimize medical treatment, that should be titrated based on a careful risk/benefit evaluation. Avoiding known triggers and eliciting habits is a milestone of counseling for pregnant women. H1 antihistamines dexchlorpheniramine, cetirizine, and loratadine, as well as the H2 antihistamine ranitidine, are considered to have good safety profiles in pregnancy. There are more concerns about the use of steroids, especially in the first trimester.

Clinicians should be aware of risk of preterm labor, potentially elicitated by MC degranulation; in this regard H1 and H2 antihistamines, cromolyn e MC stabilizer should be considered for the treatment of preterm labor.

The risk of occurrence of a severe intrapartum MC-related reaction is significantly increased during labor and delivery in comparison with the whole pregnancy, and this can be due to anesthetic procedures, other drugs eventually used, and the labor stress itself, and therefore women with mastocytosis must be adequately managed.

Predelivery planning can help and prepare the staff and the patients for complications. Despite there is not a clear evidence of efficacy, it seems reasonable to administer premedication with H1 and H2 antihistamines and with benzodiazepine to reduce anxiety levels to all pregnant mastocytosis patients during the initiation of labor. A good pain control with anesthetic procedures appear to be safe and may contribute to reduce the risk of MC degranulation and exacerbation of the disease.

As a preventive measure, resuscitation equipment should be available throughout during labor, delivery and the postpartum period, to treat unanticipated hypotension and shock.

## Unmet Clinical Needs and Futures Perspectives

Pregnancy in mastocytosis patients represents a double challenge: the goals to be reached are both safety for the embryo (drug toxicity, obstetric complications), and for the mother (worsening of clinical manifestations). Therefore, the evaluation of fertility and the management of pregnancy in such patients presents specific issues and therapeutic challenges for both patients and physicians.

The available knowledge derives from single case reports and a few case series: the data from the literature have been summarized in **Table 3**.

**Table 3:** The course of pregnancy in patients with mastocytosis – published studies and case reports.

References, n	Women	Type of Mastocytosis	History of infertility	Pregnancies	Deliveries	Miscarriage	Complications		Type of partum	Mediator related symptoms			Children with mastocytosis
							During pregnancy	During partum		Worse	Constant	Improve	
Worobec et al.	8	5 CM, 3 ISM	2 (25)	13	11 (85)	2 (15)	1 preeclampsia, 3 LBW, 1 hydrocephalus at birth and later a developmental delay	1 Transient acceleration of fetal heart rate during labor, 1 excessive bleeding after cesarean delivery.	NA	5 (63)	NA	NA	0
Bruns et al.	12	NA	NA	12	12 (100)	NA	0	0	NA	4 (34)	7 (58)	1 (8)	NA
Matito et al.	30	4 CM, 25 ISM, 1 WDSM	1 (3)	45	45 (100*)	0*	4 anaphylaxis (1 HVA, 3 idiopathic), 3 preterm delivery, 4 LBW, 2 neonatal respiratory distress	5 MC mediator release symptoms	35 vaginal, 10 caesarean	10 (22)	20 (45)	15 (33)	1 CM
Maatouk et al. (38)	2	1 CM, 1 ISM	0	3	3 (100)	0	0	0	3 vaginal	0	NA	NA	NA
Ciach et al	17	7 CM, 10 ISM	0	23	17 (74)	6 (26)	1 preeclampsia, 4 preterm delivery, 3 LBW, 2 preterm labor,1 deep vein thrombosis, 1 toxoplasmosis	0	12 vaginal, 5 caesarean	4 (24)	13 (76)	0	1 CM
Donahue et al.	1	CM (TMEP)	0	4	2 (50)	2 (50)	1 preeclampsia, 2 preterm delivery, 1 anaphylactoid reaction,1 vaginal bleeding	0	1 vaginal, 1 caesarean	2 (100)	0	0	0
Kehoe et al.	1	ISM	0	1	1 (100)	0	Preterm labor	0	Vaginal	0	1 (100)	0	0
Madendag et al.	1	CM	0	1	1 (100)	0	Preterm labor	0	Vaginal	1 (100)	0	0	0
Watson et al.	1	MIS	0	7	2 (29)	5 (71)	2 preterm delivery, 1 anaphylaxis	1 anaphylaxis	1 vaginal, 1 caesarean	NA	NA	NA	0
Villeneuve et al. (39)	1	CM (UP)	0	1	1 (100)	0	0	0	Caesarean	0	0	0	0
Garcia et al. (40)	1	ISM	NA	1	1 (100)	NA	NA	NA	NA	NA	NA	NA	NA
Gupta et al. (41)	1	CM (UP)	0	1	1 (100)	0	0	0	Vaginal	NA	NA	NA	0
Lei et al	1	ISM	0	1	1 (100)	0	1 fetal bradycardia	0	Cesarean	0	1 (100)	0	0

*Study analyzed only term pregnancies, 6 miscarriages reported out of 51 pregnancies before selection of study population.

UP, urticaria pigmentosa; TMEP, teleangectasia maculare eruptiva perstans; MIS, Mastocytosis in the skin; NA, Not Available.

A diagnosis of SM does not globally appear to affect fertility, although published data are very scarce and derived from retrospective observational studies. It was demonstrated that normal MC play an important physiological role in reproductive function, and therefore an extensive prospective evaluation of young women affected by SM in terms of ovarian function and fertility potential would be interesting and could help physicians to adequately counsel patients about their desire of conception.

The clinical manifestations of mastocytosis during pregnancy can be highly variable, as patients can experience a significant improvement of their health’s status with reduction in frequency and intensity of MC mediator-release symptoms, or can suffer by a worsening of symptoms. Moreover, the severity of symptoms can be different in the same patient in consecutive pregnancies. Similarly, anaphylaxis can occur both in patients that have already experienced this event before becoming pregnant and in naïve patients, and it can be idiopathic or triggered by known antigens. It has been suggested that the reduction in medication and a discontinuous drug intake, as well as the hormonal and immunological changes occurring during pregnancy could explain the variability of mastocytosis symptoms. Nevertheless, to date there are no data about serial monitoring of serum tryptase and other MC mediators or hormonal dosages during pregnancy. In addition, some molecules secreted by the placenta, some of them with immunomodulating properties, may also affect MC activity and therefore play a role in the mastocytosis symptoms’ evolution during pregnancy.

It would be relevant to prospectively evaluate potential correlations between clinical and biological parameters, such as the presence CKIT mutation, the degree of bone marrow infiltration, the presence of skin lesions, an history of anaphylaxis or osteoporosis, and if these features can influence the outcome of the pregnancy.

Whether mastocytosis can result in significantly increased rates of spontaneous miscarriage or obstetric complications is still unclear. Available data derive from single case report or small case series. Surely, clinicians should be aware of the risk of preterm labor and birth. The inclusion of the worldwide available data on pregnancy and fertility issues in a disease-specific registry would provide a useful support in clinical decision making and in communication with patients. An international multicenter prospective survey of pregnancies in women with mastocytosis could help to establish the relative risk of pregnancy complications in comparison with the general population. A longer follow-up of the babies, possibly extended to adulthood, would provide more precise information about the incidence of familial mastocytosis.

## Author Contributions

All the authors contributed to the critical appraisal of literature, to manuscript conception and writing. All the authors approved the final version of the manuscript.

## Conflict of Interest

The authors declare that the research was conducted in the absence of any commercial or financial relationships that could be construed as a potential conflict of interest.

## Publisher’s Note

All claims expressed in this article are solely those of the authors and do not necessarily represent those of their affiliated organizations, or those of the publisher, the editors and the reviewers. Any product that may be evaluated in this article, or claim that may be made by its manufacturer, is not guaranteed or endorsed by the publisher.
